# Organization of the Zone of Transition between the Pretectum and the Thalamus, with Emphasis on the Pretectothalamic Lamina

**DOI:** 10.3389/fnana.2016.00082

**Published:** 2016-08-11

**Authors:** Emmanuel Márquez-Legorreta, José de Anchieta C. Horta-Júnior, Albert S. Berrebi, Enrique Saldaña

**Affiliations:** ^1^Neuroscience Institute of Castilla y León (INCyL), University of SalamancaSalamanca, Spain; ^2^Department of Cell Biology and Pathology, Medical School, University of SalamancaSalamanca, Spain; ^3^Department of Anatomy, Institute of Biosciences of Botucatu, UNESP—Universidade Estadual PaulistaSão Paulo, Brazil; ^4^Department of Otolaryngology–Head and Neck Surgery and the Sensory Neuroscience Research Center, West Virginia UniversityMorgantown, WV, USA; ^5^Institute of Biomedical Research of Salamanca (IBSAL), University of SalamancaSalamanca, Spain

**Keywords:** auditory thalamus, posterior limitans nucleus of the thalamus, suprageniculate nucleus, medial geniculate body, anterior pretectal nucleus, ethmoid nucleus, *Wisteria floribunda* agglutinin (WFA), rat

## Abstract

The zone of transition between the pretectum, derived from prosomere 1, and the thalamus, derived from prosomere 2, is structurally complex and its understanding has been hampered by cytoarchitectural and terminological confusion. Herein, using a battery of complementary morphological approaches, including cytoarchitecture, myeloarchitecture and the expression of molecular markers, we pinpoint the features or combination of features that best characterize each nucleus of the pretectothalamic transitional zone of the rat. Our results reveal useful morphological criteria to identify and delineate, with unprecedented precision, several [mostly auditory] nuclei of the posterior group of the thalamus, namely the pretectothalamic lamina (PTL; formerly known as the posterior limitans nucleus), the medial division of the medial geniculate body (MGBm), the suprageniculate nucleus (SG), and the ethmoid, posterior triangular and posterior nuclei of the thalamus. The PTL is a sparsely-celled and fiber rich flattened nucleus apposed to the lateral surface of the anterior pretectal nucleus (APT) that marks the border between the pretectum and the thalamus; this structure stains selectively with the *Wisteria floribunda* agglutinin (WFA), and is essentially immunonegative for the calcium binding protein parvalbumin (PV). The MGBm, located medial to the ventral division of the MGB (MGBv), can be unequivocally identified by the large size of many of its neurons, its dark immunostaining for PV, and its rather selective staining for WFA. The SG, which extends for a considerable caudorostral distance and deviates progressively from the MGB, is characterized by its peculiar cytoarchitecture, the paucity of myelinated fibers, and the conspicuous absence of staining for calretinin (CR); indeed, in many CR-stained sections, the SG stands out as a blank spot. Because most of these nuclei are small and show unique anatomical relationships, the information provided in this article will facilitate the interpretation of the results of experimental manipulations aimed at the auditory thalamus and improve the design of future investigations. Moreover, the previously neglected proximity between the MGBm and the caudal region of the scarcely known PTL raises the possibility that certain features or roles traditionally attributed to the MGBm may actually belong to the PTL.

## Introduction

The zones of transition between brain regions with different embryological origins are structurally complex. A good example is the transition zone between the pretectum, derived from prosomere 1, and the thalamus, derived from prosomere 2 (Puelles et al., 2012). Whereas the most rostral cell group of the pretectum is the large and well-characterized anterior pretectal nucleus (APT; Puelles et al., 2012; Sefton et al., 2015), the caudal thalamus includes several mostly small and ill-defined nuclei, collectively known as the posterior group of the thalamus, whose size and organization seem to differ among species (Jones, 2007).

As part of our long-term goal of characterizing the connections between auditory nuclei of the mammalian brain, we are interested in the pretectothalamic zone because several nuclei of the posterior thalamus, such as the medial division of the medial geniculate body (MGBm) and the suprageniculate nucleus (SG), are well-known targets of projections from auditory centers (Linke, 1999; Mellott et al., 2014) and are considered relay stations of the so-called extralemniscal auditory pathway (e.g., Lee, 2015). The pretectothalamic zone includes also another much less studied structure that receives dense projections from the inferior colliculus (Kudo and Niimi, 1980; LeDoux et al., 1985, 1987), and therefore may participate in the processing of hitherto unknown aspects of acoustic information. This enigmatic colliculo-recipient structure, which is a fiber-rich and cell-poor lamina apposed to the lateral surface of the APT, may constitute the physical limit between the pretectum and the thalamus. Unfortunately, the nature of this fibrillary lamina is completely uncertain because its analysis has been hindered not only by its unusual shape, but also by severe terminological confusion.

The narrow colliculo-recipient area apposed to the APT was first discerned as a separate nucleus by LeDoux et al. (1985), who described its afferents from the inferior colliculus. These authors referred to it as the “posterior limitans nucleus of the thalamus (PLi)”, a term that was soon incorporated into the renowned stereotaxic atlas of the rat brain by Paxinos and Watson (1986) and later editions: e.g., Paxinos and Watson (2007). LeDoux et al. (1985, 1987) followed the nomenclature proposed by Winer and Morest (1983a,b) for the auditory thalamus of the cat. Interestingly, the structure Winer and Morest (1983a,b) called the “posterior limitans nucleus” does not seem to correspond to the pretectothalamic border itself, but to a small cell group located in the most medial part of the dorsal division of the MGB (MGBd), and hence lateral to the border. Winer and Morest (1983a,b) interpreted this cell group of the MGBd as the caudal extension of the classical “limitans nucleus” (Friedemann, 1911), a well-recognized thalamic structure best characterized in primates whose cytoarchitecture differs markedly from that of the laminar pretectothalamic border (Moryś and Mamos, 1987; Moryś et al., 1989; Jones, 2007). This brief retrospective emphasizes that the zone of transition between the pretectum and the thalamus of mammals contains three structures that, despite being truly different, bear very similar names: (1) The [classical] limitans nucleus of the thalamus, located lateral to the fibrillary pretectothalamic border (Moryś et al., 1989; Jones, 2007); (2) the posterior limitans nucleus of Winer and Morest (1983a,b), which is also lateral to the pretectothalamic border; (3) and the PLi (LeDoux et al., 1985, 1987; Paxinos and Watson, 2007), which coincides with the pretectothalamic border itself.

To clarify the organization of the zone of transition between the pretectum and the thalamus of the rat, we performed the first comprehensive and multitechnical morphological investigation of the pretectothalamic border. We have also refined the nomenclature of this complex diencephalic region by referring to the colliculo-recipient fiber-rich border formerly known as the PLi with the descriptive name “pretectothalamic lamina” (PTL); this name is based on a previous proposal by Moryś and coworkers (Moryś and Mamos, 1987; Moryś et al., 1987, 1989; Słoniewski et al., 1987). Our results reveal several morphological criteria useful to define the PTL and its neighboring nuclei. Given the peculiar shape of the PTL and the small size of most nuclei of the caudal thalamus, this information will be essential to interpret the results of future investigations, as experimental manipulations aimed at any one given structure of this area are likely to affect nearby structures as well.

## Materials and Methods

We have used a panel of morphological techniques to describe the organization of the zone of transition between the pretectum and the thalamus of the rat. Applied techniques include analysis of fresh, unstained sections; basic neurohistological procedures (Nissl method and Giemsa method); myelin staining with osmium tetroxide; immunocytochemistry using antisera directed against the calcium binding proteins parvalbumin (PV), calbindin (CB) and calretinin (CR); and histochemistry for *Wisteria floribunda* agglutinin (WFA) and acetylcholinesterase (AChE).

### Experimental Animals

Adult female Wistar rats (body weight 190–290 g) were cared for and used in compliance with European Union regulations concerning the use of animals in biomedical research. The experimental procedures were approved and supervised by the Bioethics Committee of the University of Salamanca. For the transcardial perfusion of fixatives, the animals were deeply anesthetized with an overdose of sodium-pentobarbital administered intraperitoneally.

### Cytoarchitectural Analysis

We studied 40–60 μm thick frozen sections or 15 μm thick paraffin-embedded sections of the rat brain stained with either the Nissl method (0.25–1% cresyl violet) or the Giemsa method (Íñiguez et al., 1985). This material was available from the histological collection of our laboratory at the University of Salamanca.

### Myeloarchitectural Analysis

We examined 60 μm thick brainstem sections postfixed with OsO_4_. To produce them, we perfused two young adult rats with 2.5% glutaraldehyde and 2% formaldehyde (prepared from freshly depolymerized paraformaldehyde), dissected the brains and left them in the same fixative overnight. The brains were sectioned with a Vibratome at a thickness of 60 μm. Sections were postfixed in ice-chilled 1% OsO_4_ and 5% sucrose in phosphate buffer for about 1 h, dehydrated, cleared with xylene, mounted on slides and coverslipped with Entellan (Merck, Darmstadt, Germany).

We also examined 40 μm thick sections of formaldehyde-fixed brains that were photographed in the unstained and uncleared state immediately after sectioning. For this purpose, we perfused young adult rats with 4% formaldehyde (prepared from freshly depolymerized paraformaldehyde or from commercial formalin), dissected the brains, cryoprotected them in 30% sucrose and sectioned them with a freezing microtome. The 40 μm thick sections were allowed to thaw free-floating, transferred to a microscopic slide, coverslipped and photographed unstained in an aqueous medium. The contrast of the micrographs was enhanced uniformly using the Auto Contrast tool of Adobe Photoshop (Adobe, San Jose, CA, USA) software. For convenience, throughout the article, we will refer to these wet unprocessed sections as “fresh sections” (Aparicio and Saldaña, 2014). Once photographed, these sections were processed immunocytochemically to visualize calcium binding proteins (see below).

### Immunostaining for Calcium Binding Proteins

We visualized the calcium binding proteins PV, CB and CR in free floating 40 μm thick frozen sections of five formaldehyde fixed brains. For each brain, adjacent sections were stained for PV, CB, CR and Nissl (cresyl violet), so that the distribution of each marker could be analyzed in 25% of the sections. The protocol to visualize PV included incubation of the sections in a mouse anti-PV primary antiserum (1:8000–1:300; ref. P3088 of Sigma, Saint Louis, MO, USA), followed by biotinylated anti-mouse immunoglobulin G raised in goat (1:200; Sigma, ref. B0529). For CB we incubated the sections in a monoclonal anti-CB D-28K antibody produced in mouse (1:500; Sigma, ref. C9348), and then in biotinylated anti-mouse immunoglobulin G raised in goat (1:200; Sigma, ref. B0529). To visualize CR, the sections were incubated in an anti-CR polyclonal antiserum produced in rabbit (1:500; Sigma, ref. C7479) and then in biotinylated anti-rabbit immunoglobulin G raised in goat (1:200; ref. BA-1000 of Vector Labs, Burlingame, CA, USA). Following the incubation in the secondary antiserum, the sections were processed by the avidin–biotin–peroxidase complex procedure (ABC; Vectastain, Vector) following the manufacturer’s specifications, and then by standard histochemistry for peroxidase, with or without heavy-metal intensification (i.e., López et al., 1999).

### Histochemistry for *Wisteria floribunda* Agglutinin and Acetylcholinesterase

To visualize perineuronal nets (Seeger et al., 1994), three rats were perfused with 4% formaldehyde (prepared from freshly depolymerized paraformaldehyde) and the brains were dissected, cryoprotected in 30% sucrose and sectioned with a freezing microtome at a thickness of 40 μm. Every other section was stained with biotinylated *WFA* (Sigma, ref. L-1766). For comparative analysis and cytoarchitectural reference, the other sections were processed with the standard histochemical procedure of Karnovsky and Roots (1964) to visualize the enzyme AChE. For the WFA technique, we followed the protocol published by Härtig et al. (1995). Free-floating sections were rinsed with 0.1 M Tris-buffered saline (TBS), pH 7.4, endogenous peroxidase activity was abolished with diluted hydrogen peroxide (H_2_O_2_), and the non-specific binding sites were blocked with 2% bovine serum albumin in TBS (TBS-BSA). Sections were then incubated with biotinylated WFA (2 μg/ml in TBS-BSA), overnight at 4°C, and then in the ABC complex. Finally, the peroxidase bound to the lectin was demonstrated by incubating the sections for 15 min, under light microscopic control, in a nickel-enhanced diaminobenzidine solution containing 0.4% nickel ammonium sulfate, 0.016% diaminobenzidine tetrahydrochloride and 0.03% H_2_O_2_ in 0.05 M Tris, pH 8.0, resulting in a black reaction product. A subset of the sections stained for WFA or for AChE were counterstained with cresyl violet or neutral red. All sections were mounted onto glass slides, dehydrated, cleared and coverslipped with Entellan (Merck).

### Reference Maps

**Figure 1:**
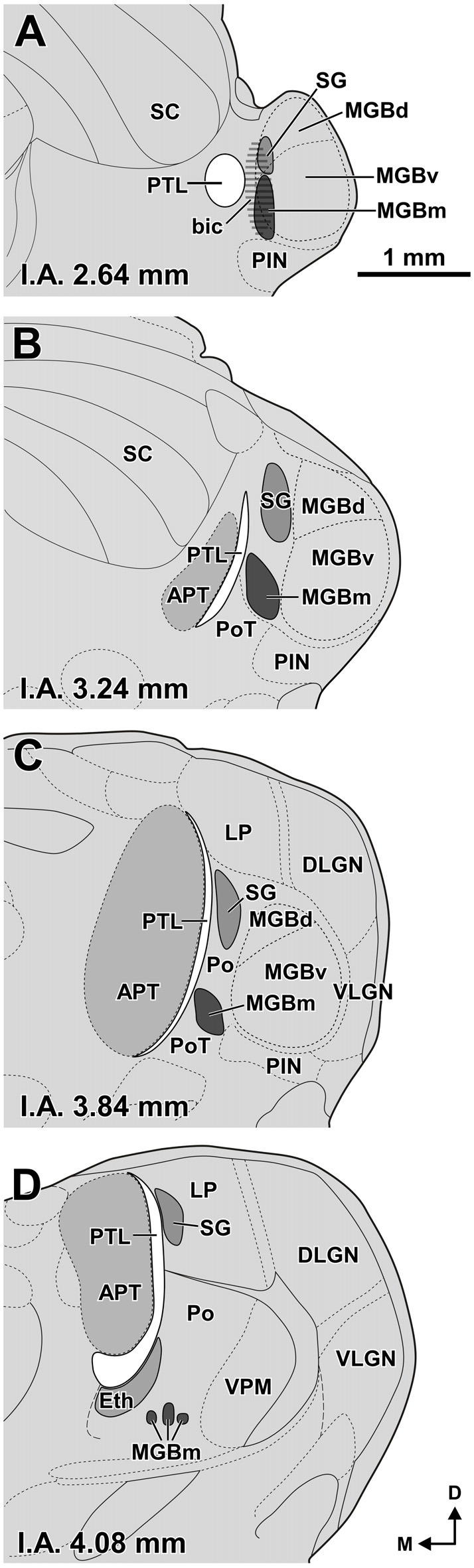
**Size and position of nuclei in the zone of transition between the pretectum and the thalamus of the rat. (A–D)** Schematic drawings of four idealized coronal sections. The number at the bottom of each schematic indicates the distance in millimeters between the depicted plane and the interaural (I.A.) coronal plane. The pretectothalamic lamina (PTL), formerly known as the posterior limitans nucleus of the thalamus (PLi), illustrated in white, is largely apposed to the lateral aspect of the anterior pretectal nucleus (APT) and constitutes a physical pretectothalamic border. The medial division of the medial geniculate body (MGBm) occupies a position medial to the ventral division of the MGB (MGBv). The stippling in **(A)** represents the brachium of the inferior colliculus (bic). The suprageniculate nucleus (SG) extends rostrally and dorsally beyond the level of the MGB approaching the dorsal end of the PTL. The ethmoid nucleus (Eth) is apposed to the ventrolateral aspect of the rostral PTL. Other abbreviations: DLGN, dorsolateral geniculate nucleus; LP, lateral posterior thalamic nucleus; MGBd, dorsal division of the MGB; PIN, posterior intralaminar nucleus; Po, posterior nucleus of the thalamus; PoT, posterior triangular nucleus of the thalamus; SC, superior colliculus; VLGN, ventrolateral geniculate nucleus, VPM, ventral posteromedial thalamic nucleus. Calibration bar in **(A)** applies to all drawings.

Initially, we adopted the parcellation of the auditory thalamus of the rat as depicted in the atlas of Paxinos and Watson (2007), which is based on the work of LeDoux et al. (1985, 1987). However, as will become evident throughout the article, our results have led to a refined scheme of the organization of the auditory thalamus, which is shown in Figure 1.

### Photography and Illustrations

**Figure 2:**
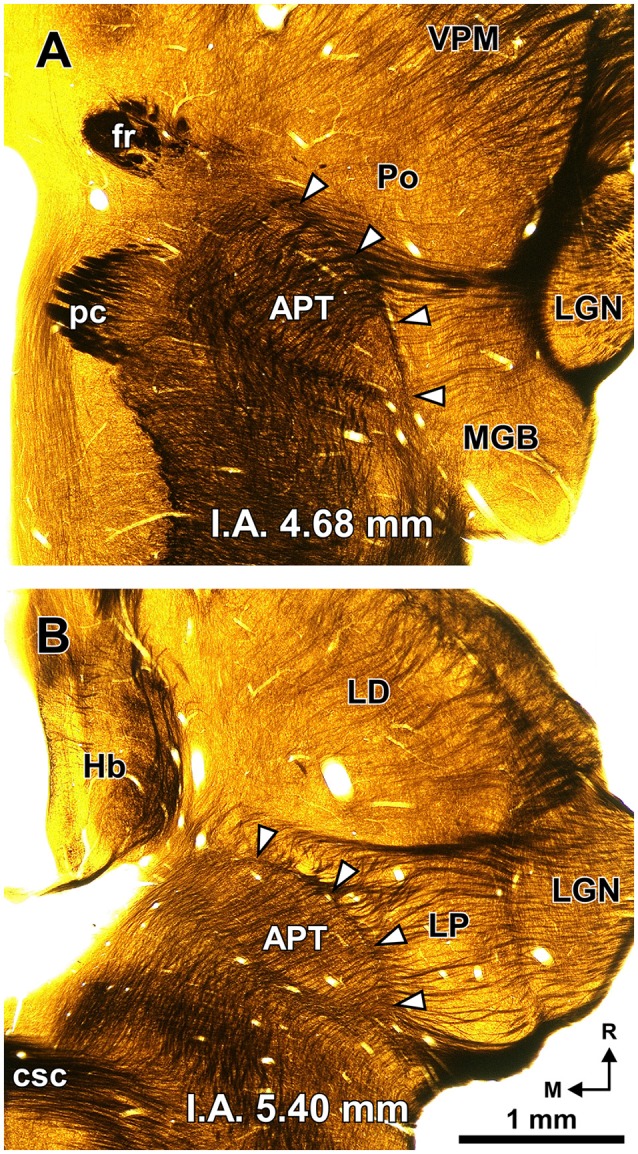
**The PTL is a natural border between the pretectum and the thalamus.** Semi-panoramic view of the region of transition between the pretectum and the thalamus of the rat as seen in two 60 μm thick horizontal sections postfixed with osmium tetroxide to stain myelinated fibers black. The white arrowheads point to the PTL, which stands out as a fiber rich sheet closely apposed to the lateral and rostral border of the APT. The distance in millimeters between the depicted section and the I.A. horizontal plane is indicated at the bottom of the micrographs. Calibration bar in **(B)** applies also to **(A)**. Other abbreviations: csc, commissure of the superior colliculus; fr, fasciculus retroflexus; Hb, habenula; LD; laterodorsal nucleus of the thalamus; LGN, lateral geniculate nucleus; LP, lateral posterior thalamic nucleus; pc, posterior commissure; Po, posterior nucleus of the thalamus; VPM, ventral posteromedial thalamic nucleus.

**Figure 3:**
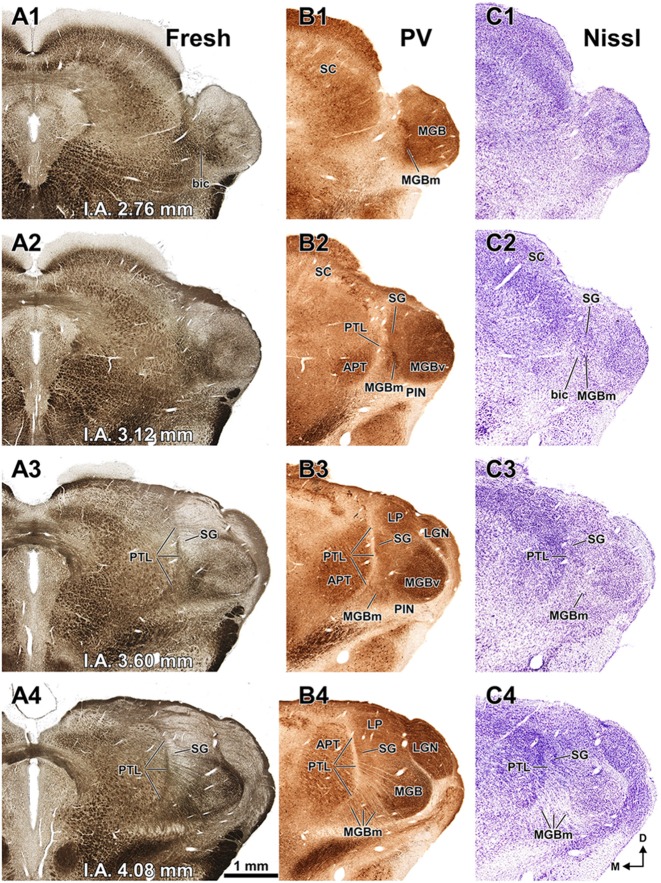

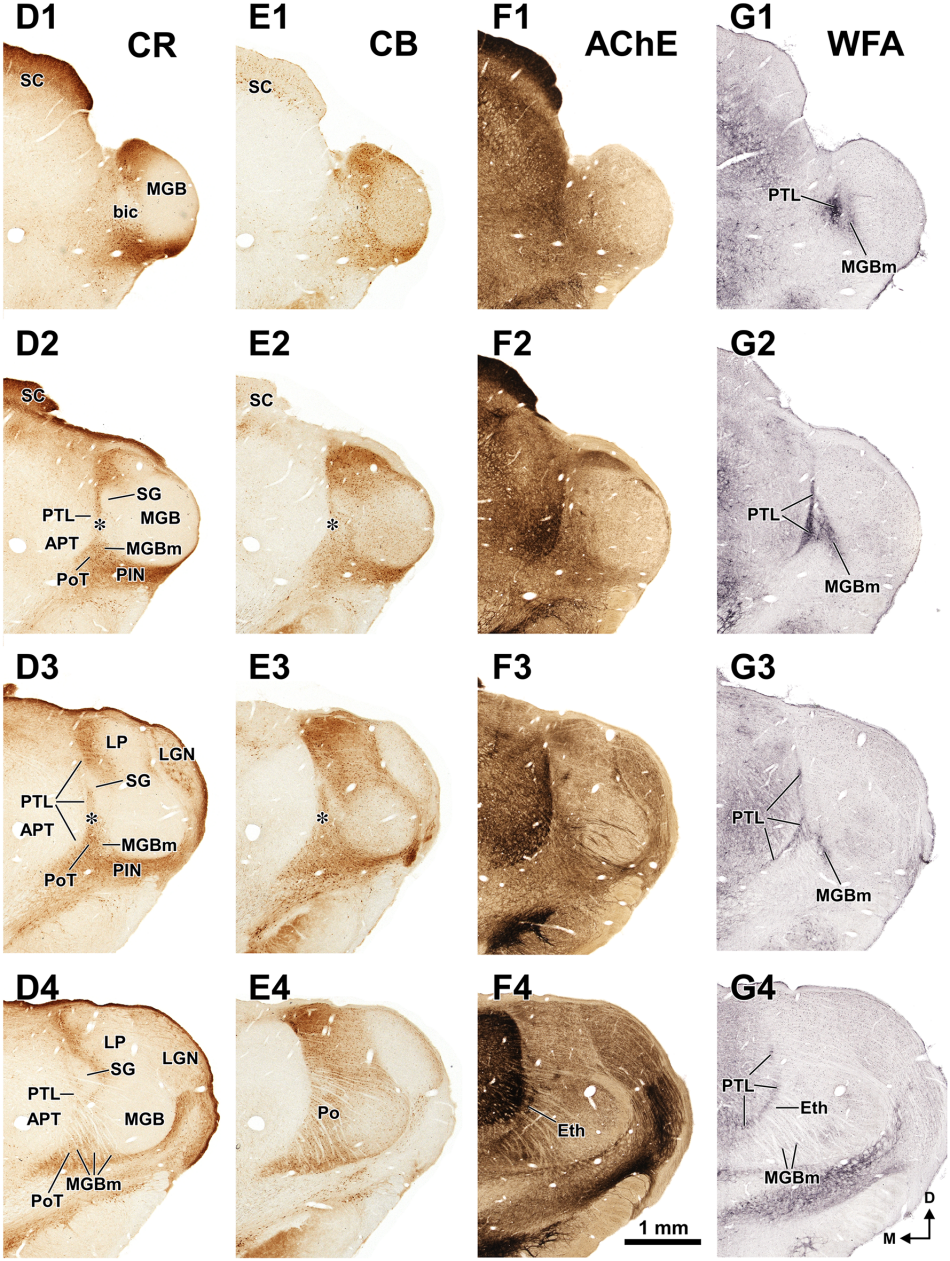
**The pretectum and posterior thalamus of the rat in coronal sections.** Micrographs of coronal sections through various rostrocaudal levels of the zone of transition between the pretectum and the thalamus. All micrographs in any given row correspond to the same rostrocaudal level, whose distance with respect to the I.A. is indicated in column **(A)**. Column **(A)**: fresh unstained and uncleared sections. Cross-cut myelinated axons appear dark, thus delineating the PTL and other myelin-rich territories and fascicles. Column **(B)**: the same sections shown in **(A)**, photographed after immunostaining for parvalbumin (PV). Notice that the heavily myelinated PTL visible in **(A)** stains very lightly for PV. A higher magnification of **(B3)** is shown in Figure 5A. Column **(C)**: sections adjacent to those of column **(B)** stained with cresyl violet. A higher magnification of **(C3)** is shown in Figure 5B. Calibration bar in **(A4)** applies to all micrographs in columns **(A–C)**. Column **(D)**: sections immunostained for calretinin (CR). Notice the absence of labeling in the PTL, SG and most of the MGB. The asterisk in **(D2,D3)** indicates an immunoreactive vertical stripe immediately lateral to the PTL. A higher magnification of **(D3)** is shown in Figure 4B. Column **(E)**: sections adjacent to those in column **(D)** immunostained for calbindin (CB). Notice the absence of labeling in the PTL and the APT, and the very weak labeling in SG. The asterisk in **(E2,E3)** indicates an immunoreactive vertical stripe immediately lateral to the PTL. A higher magnification of **(E3)** is shown in Figure 4C. Column **(F)**: sections stained for acetylcholinesterase (AChE). Notice the very dense staining of APT, which contrasts with the much lighter PTL. A higher magnification of **(F3)** is shown in Figure 5C. Column **(G)**: sections adjacent to those of column **(F)** stained for *Wisteria floribunda* agglutinin (WFA). Notice the selective staining of the PTL and the MGBm. A higher magnification of **(G3)** is shown in Figure 5D. Calibration bar in **(F4)** applies to all micrographs in columns **(D–G)**. Other abbreviations: bic, brachium of the inferior colliculus; Eth, ethmoid thalamic nucleus; LGN, lateral geniculate nucleus; LP, lateral posterior thalamic nucleus; MGB, medial geniculate body; MGBm, medial division of MGB; PIN, posterior intralaminar nucleus; Po, posterior thalamic nucleus; PoT, posterior triangular nucleus of the thalamus; SC, superior colliculus.

**Figure 4:**
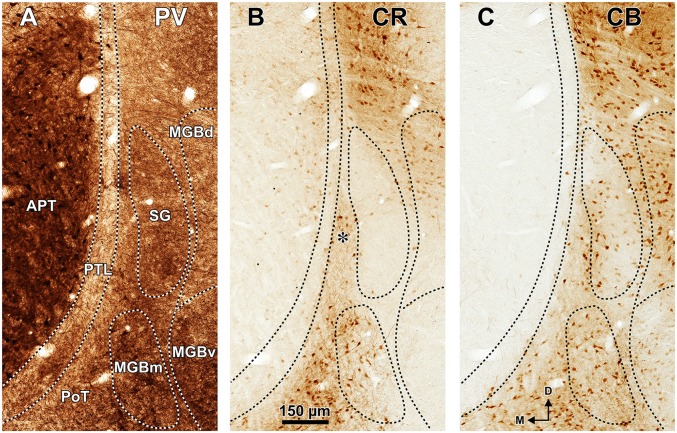
**Distribution of calcium binding proteins in the zone of transition between the pretectum and the thalamus of the rat.** Micrographs of three adjacent coronal sections through the center of the PTL. The PTL stains weakly for PV **(A)**, and is for the most part devoid of CR **(B)** and CB **(C)**. Notice that the APT is strongly immunopositive for PV **(A)** and the SG is conspicuously immunonegative for CR **(B). (B,C)** Show details of the sections depicted in Figures 3D3,E3. The asterisk in **(B)** indicates an immunoreactive vertical stripe immediately lateral to the PTL. Other abbreviations: MGBd, dorsal division of the medial geniculate body; MGBm, medial division of the medial geniculate body; MGBv, ventral division of the medial geniculate body; PoT, posterior triangular nucleus of the thalamus. Calibration bar in **(B)** applies to all micrographs.

**Figure 5:**
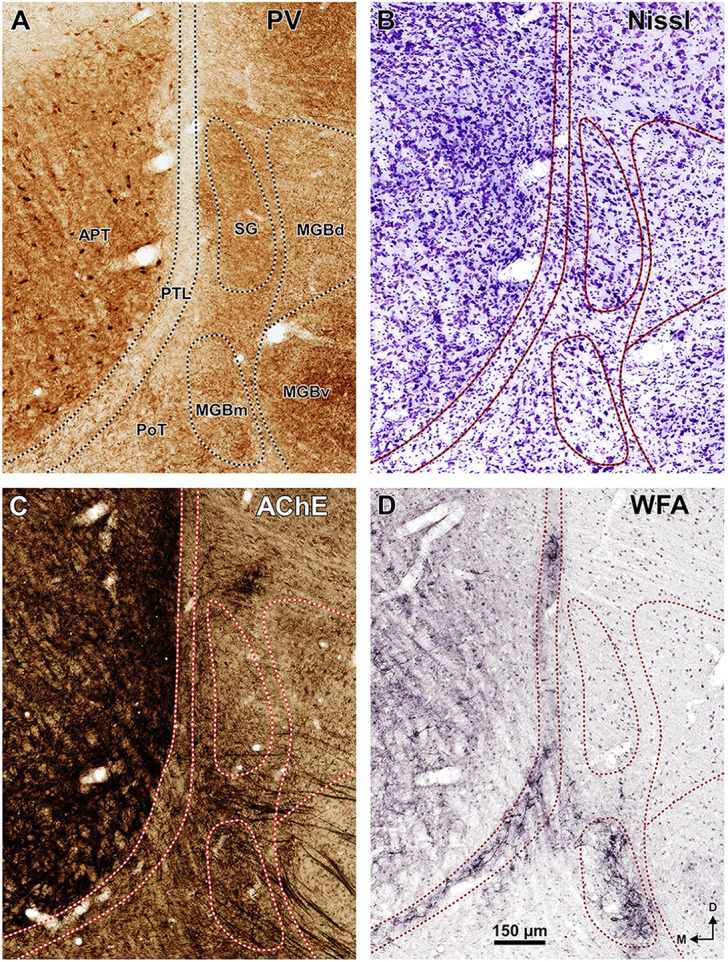
**Neurochemical features of the zone of transition between the pretectum and the thalamus of the rat.** Micrographs of coronal sections through the center of the PTL stained for PV **(A)**, Nissl **(B)**, AChE **(C)**, and *WFA* **(D)**. The PTL stains weakly for PV **(A)** and AChE **(C)**, but is highlighted by the positive staining with WFA **(D)**. Moreover, the PTL is characterized by its low neuronal density **(B)**. The MGBm is also highlighted with WFA **(D)**. Section **(A)** (shown at lower magnification in Figure 3B) is adjacent to section **(B**; shown in Figure 3C3**)**, and section **(C**; shown in Figure 3G3**)** is adjacent to section **(D**; shown in Figure 3F3**)**. Other abbreviations: MGBd, dorsal division of the medial geniculate body; MGBv, ventral division of the MGB; PoT, posterior triangular nucleus of the thalamus. Calibration bar in **(D)** applies to all micrographs.

**Figure 6:**
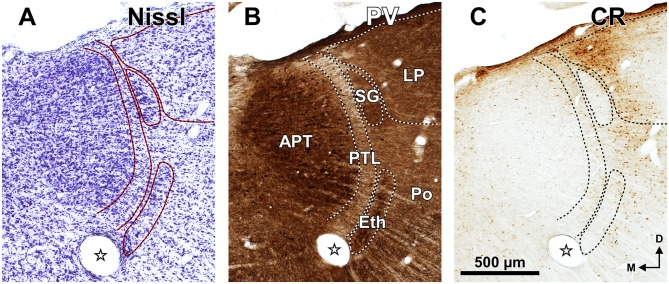
**The zone of transition between the pretectum and the thalamus of the rat in rostral coronal sections.** Micrographs of three adjacent sections through the rostral portion of the APT, PTL and SG stained for the Nissl method **(A)**, PV **(B)**, and CR **(C)**. Notice the high neuronal density and strong PV immunoreactivity in APT, SG and the ethmoid nucleus (Eth). The SG and Eth are virtually devoid of CR **(C)**. The stars indicate a fiducial mark made to align the sections. Other abbreviations: LP, lateral posterior thalamic nucleus; Po, posterior thalamic nucleus. Calibration bar in **(C)** applies also to **(A,B)**.

**Figure 7:**
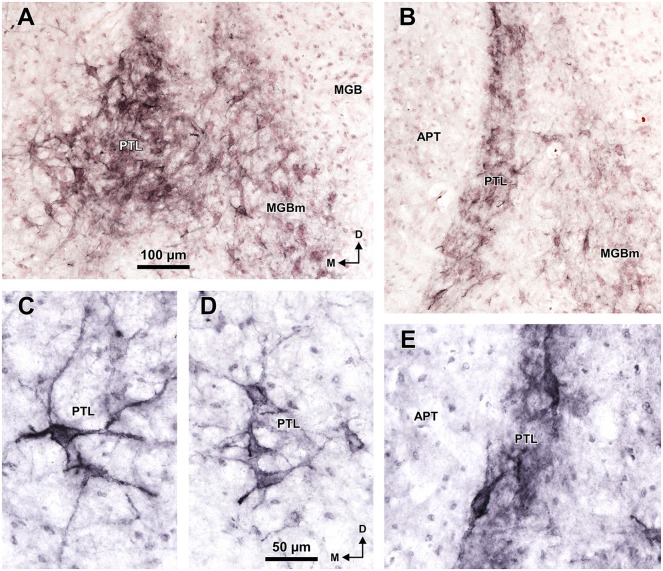
**Selective staining of the PTL and MGBm of the rat with *WFA*.** Micrographs of coronal sections through the caudal **(A,C,D)** and central portions **(B,E)** of the PTL. At caudal levels, the darker staining and higher concentration of perineuronal nets in the PTL distinguishes it clearly from the lighter MGBm **(A)**. The WFA-positive perineuronal nets reveal that PTL neurons are predominantly bipolar in the central portions of the nucleus **(B,E)**, and mostly multipolar in the most caudal sections **(A,C,D)**. The sections shown in **(A,B)** are counterstained with neutral red. Other abbreviations: APT, anterior pretectal nucleus; MGB, medial geniculate body. The calibration bar in **(A)** applies also to **(B)**, and the calibration bar in **(D)** applies also to **(C,E)**.

**Figure 8:**
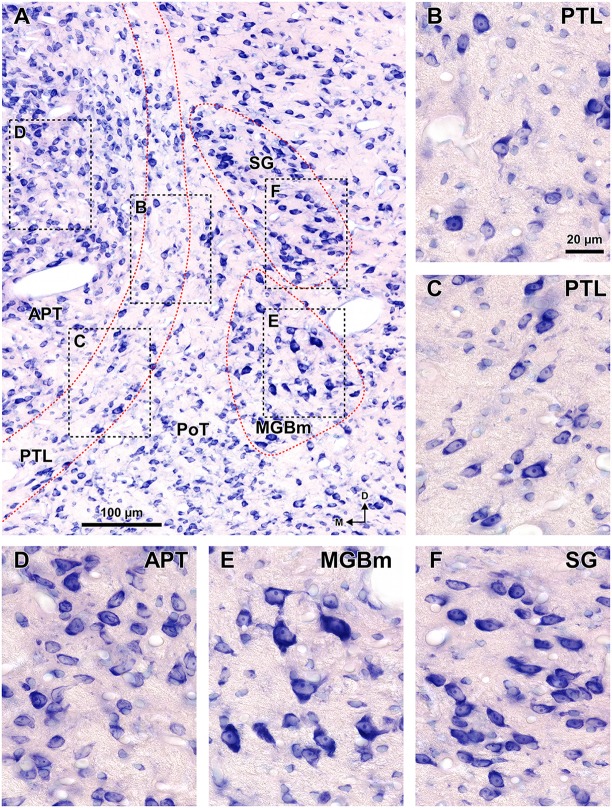
**Cytoarchitecture of the zone of transition between the pretectum and the thalamus of the rat.** Micrographs of a 15 μm thick paraffin embedded coronal section through the mid-rostrocaudal level of the transition zone stained by the Giemsa method. **(A)** Semipanoramic view. The rectangular areas framed by black dotted lines are shown at higher magnification in **(B)** through **(F). (B,C)** The neurons of the PTL are small and their bipolar cell bodies are oriented parallel to the outline of the nucleus. **(D)** The heterogeneous, tightly packed neurons of the APT lack a preferential orientation. **(E)** The MGBm includes large scattered neurons. **(F)** The SG consists of rather homogeneous and tightly packed neurons, many of them oriented parallel to each other. Other abbreviations: PoT, posterior triangular nucleus of the thalamus. Calibration bar in **(B)** applies also to **(C–F)**.

The sections shown in Figures 3–8 were photographed at high resolution with a Zeiss Axioskop 40 microscope using a Zeiss AxioCam MRc 5 digital camera (Carl Zeiss, Oberkochen, Germany). The thick sections postfixed with OsO_4_ depicted in Figure 2 were photographed with a Leica M165 FC stereomicroscope equipped with a Leica DFC500 digital camera. The brightness and contrast of images were adjusted uniformly with Adobe Photoshop software, and the illustrations were arranged into plates using Canvas (ACD Systems of America, Inc., Miami, FL, USA) software.

The sections shown in Figures 3B,C underwent an 11% linear shrinkage following histological processing. Therefore, the micrographs of these sections were scaled up by 11% to match the size of the fresh sections shown in Figure 3A.

## Results

Our panel of morphological techniques indicates that the region of transition between the pretectum and the thalamus of the rat includes the following structures (ordered from caudal to rostral): (a) the APT; (b) the PTL, which constitutes the actual pretectothalamic border; (c) the most caudal nuclei of the posterior thalamus, including the MGBm, the SG, the ethmoid nucleus (Eth), the posterior triangular nucleus (PoT), and the posterior nucleus (Po); and (d) the lateral posterior nucleus (LP), which belongs to the lateral complex of the thalamus, but is adjacent to the most rostrodorsal aspect of the PTL. These structures, whose size and position are illustrated schematically in Figure 1, are described in the following sections. We first provide a brief statement of the main cyto-, chemo- and/or myelo-architectural features useful to locate and identify each nucleus, followed by more detailed explanations. Special emphasis is placed on the PTL.

### Anterior Pretectal Nucleus (APT)

The APT is unmistakable due to its high neuronal density, moderate degree of myelination, very strong staining for PV AChE, and lack of staining for CB (Figures 2–6). CR is present in only a few neurons of the dorsolateral region of the nucleus, and these are weakly stained (Figure 4B). In sections stained for *WFA* the nucleus shows weak or moderate diffuse labeling (Figures 3G3, 5D); however, the lateral region of APT (a stripe 100–200 μm thick) is conspicuously devoid of labeling, thus providing a sharp contrast with the strongly labeled PTL (Figures 3G, 5D, 7B,E). Very few APT neurons are delineated by WFA-stained perineuronal nets, and these are always found at a distance from the PTL (Figures 3G, 5D).

The APT has been studied in multiple species, including rat (e.g., Bokor et al., 2005; Giber et al., 2008). Therefore, it will not be considered further in this article, except when it constitutes a useful anatomical reference for other structures.

### Pretectothalamic Lamina (PTL)

The PTL is characterized by its high density of myelinated fibers, strong selective labeling with WFA, and low expression or absence of PV, CR, CB and AChE (Figures 2–7). Moreover, PTL neurons are scattered among myelinated fibers and display distinct cytological features (Figure 8).

The rat PTL is a narrow and vertical nucleus oriented rostrocaudally and apposed to the lateral and rostral surface of the APT (Figure 1). Its extent coincides for the most part with that of the APT, although its caudal pole extends beyond the caudal end of APT alongside the medial border of the MGB and the brachium of the inferior colliculus (bic; Figure 1A). In its entirety, the PTL extends rostrocaudally for approximately 2 mm, and dorsoventrally for approximately 2.5 mm. The caudal and rostral ends of the PTL correspond, respectively, to the most ventral and the most dorsal portions of the nucleus. In individual coronal sections the PTL appears as a vertical bow with its concavity open medially, and in sections through the center of the nucleus its maximum height approaches 2 mm. At most levels the nucleus is only 50–70 μm thick, but it becomes somewhat thicker ventrally, rostrally and caudally.

A fundamental criterion to identify the PTL is its abundance of myelinated fibers. In sections processed with osmium tetroxide, the PTL, readily visible by the dark staining (Figure 2, white arrowheads), creates a sharp border between the moderately and homogeneously myelinated APT, and the relatively myelin-poor thalamus (Figure 2). In horizontal sections through the MGB, the PTL appears oriented rostrocaudally along the lateral border of APT (Figure 2A), whereas in more dorsal sections, it appears bow-shaped and is apposed to the lateral and rostral surface of APT (Figure 2B). Thus, the PTL becomes situated more medially at progressively more dorsal and rostral levels.

Figure 3A shows a series of regularly spaced coronal sections through the posterior thalamus of the rat. These fresh sections were photographed in an aqueous medium immediately after sectioning, without any kind of staining. In this material, cross-cut myelinated fibers appear dark against an unstained background. At rostral levels (Figures 3A3,A4) the PTL stands out as a darker narrow, vertically oriented stripe located along the lateral border of the lighter APT, at a distance from the laterally located MGB. More caudally (Figures 3A1,A2), the PTL widens lateromedially, loses its laminar configuration, and approaches the medial border of the MGB. Nevertheless, the fascicles of axons of the PTL remain medial to the bic and the MGBm, and do not intermingle with them.

Figure 3B illustrates the same sections depicted in Figure 3A, which were later immunostained for PV. The weakly-stained PTL stands out as a sharply defined light stripe sandwiched between the APT and the thalamus (Figures 3B, 4A, 5A). Likewise, in sections stained for AChE, the APT appears so dark that it provides a sharp contrast with the much lighter PTL (Figures 3F, 5C).

The PTL is clearly immunonegative for CR (Figures 3D, 4B) and CB (Figures 3E, 4C). In some coronal sections of mid-rostrocaudal levels, a narrow vertical stripe immunostained for CR and CB is located medial to MGB (asterisk in Figures 3D2,D3,E2,E3), in a position that could be mistaken for the PTL. However, this vertical stripe occupies a position immediately lateral to the PTL and likely corresponds to the most medial part of the caudal PoT and Po.

A very useful marker to highlight selectively the PTL is the lectin WFA. It provides distinctive staining of the PTL, whereas the thalamus (with the exception of MGBm) and the region of the APT adjacent to the PTL remain for the most part unstained (Figures 3G, 5D). The PTL staining is particularly strong in the most caudal sections, in which the nucleus does not appear flattened, but as a more or less circular dark spot (Figures 3G1, 7A). The gap in the staining between the caudal pole of the PTL and the nucleus of the bic, which possesses conspicuous perineuronal nets surrounding large multipolar neurons (not shown), indicates that the former is not a rostral extension of the latter. At rostral levels, the WFA-labeled PTL wraps around the ventrolateral surface of the APT, separating it from the unstained Eth (Figure 3G4; see also Figure 6). This ventromedial region of the PTL seems to include the area occupied by the scaphoid nucleus of Paxinos and Watson (1986); see also plates 70–72 of Paxinos and Watson (2007), which in our material is not distinct. The WFA labeling becomes progressively weaker rostrally and dorsally and does not reach the most dorsal and rostral portions of the PTL (Figures 3G3,G4). Only a fraction of PTL neurons are sharply delineated by WFA-stained perineuronal nets (Figures 5D, 7). In some instances, the outlined cell bodies and proximal dendrites reveal fusiform neurons with oppositopolar dendrites (Figures 7B,E) and particularly in caudal sections, multipolar neurons without a preferential orientation (Figures 7A,C,D).

In sections stained by the Nissl method or by the Giemsa method, the sparsely-celled PTL contrasts with the much darker staining of the APT (Figures 3C, 5B, 6A, 8). In general, PTL somata contain very little cytoplasm, so they appear more lightly stained than those of surrounding nuclei (Figures 8A–C). When viewed in the coronal plane, PTL neurons tend to exhibit round or elongated cell bodies; the main axis of the fusiform neurons is usually parallel to the contour of the nucleus, particularly in its ventral half. PTL neuronal cell bodies are 10–15 μm long (range 7–19 μm) and approximately 7 μm thick (range 4–10 μm).

### Medial or Magnocellular Division of the Medial Geniculate Body (MGBm)

The MGBm can be unequivocally identified by the large size of many of its neurons, its dark immunostaining for PV, and its rather selective staining for WFA (Figures 3–5, 7, 8).

As pointed out by previous authors (e.g., LeDoux et al., 1985, 1987; Clerici and Coleman, 1990; Winer et al., 1999; Jones, 2007), the MGBm contains a population of large neurons that stain more darkly with classical neurohistological techniques than those of the adjacent nuclei (Figure 8). The MGBm always occupies a position medial to the ventral division of the MGB (MGBv), and its rostrocaudal length (approximately 1.7 mm) roughly coincides with that of the MGBv (Figures 1, 3). In caudal sections the MGBm appears as an oval, 400–500 μm tall and 200–250 μm wide, immediately adjacent to the MGBv and partly disrupted by the entrance of the bic (Figures 1A, 3, 7A). In progressively more rostral sections, the nucleus becomes gradually shorter vertically (300–350 μm) and wider (350 μm) and separates somewhat from the MGBv (Figures 1C, 3). In rostral sections the neurons of the MGBm appear scattered among fascicles of axons of the thalamic radiation that course in an approximately coronal plane (Figure 3C4).

The MGBm is strongly immunoreactive for PV and both the neuropil and cell bodies contribute to the staining (Figures 3B, 4A). This nucleus is also nicely highlighted by WFA, which is a very useful marker for the MGBm (Figures 3G, 5D, 7A,B). Nevertheless, the staining of the MGBm is more diffuse and lighter than that of the PTL (Figures 3G1,G2, 7A). The MGBm contains very few perineuronal nets stained with WFA (Figures 5D, 7A,B) and they seldom surround large neurons. Finally, in sections processed for CR, CB and AChE, the MGBm shows moderate labeling that does not highlight the nucleus (Figures 3D–F, 4B,C, 5C).

### Suprageniculate Nucleus (SG)

The SG can be readily identified by its peculiar cytoarchitecture, the paucity of myelinated fibers, and the conspicuous absence of staining for CR. Indeed, in many CR-stained sections, the SG stands out as a blank spot (Figures 3D2–D4, 4B, 6C).

The SG of the rat is surprisingly large. It extends caudorostrally without interruption for about 2 mm, from the caudal end of the MGB, to the level of the most dorsal and rostral PTL and APT. At caudal levels, the SG occupies a position medial and adjacent to MGBd. However, more rostrally the nucleus separates progressively from the MGB and shifts medially and dorsally to impinge upon what has traditionally been considered the most medial LP (Figures 1D, 6). In individual coronal sections, the SG appears as one or more relatively well-defined ovoid or teardrop-like cell groups adjacent to the PTL (Figures 3C, 5B, 6A, 8). In most sections the nucleus is 350–600 μm tall and 150–200 μm wide.

In sections stained for PV, the neuropil of the SG appears clearly darker than that of the PTL and somewhat darker than that of the MGBd (Figures 4A, 5A). The neurons of the SG possess more cytoplasm (which results in darker Nissl and Giemsa staining), and are more densely packed than those of adjacent nuclei (Figures 3C, 5B, 6A, 8). Moreover, SG neurons show a preferential ventrolateral to dorsomedial orientation. Finally no WFA-positive perineuronal nets are found in the SG (Figures 3G, 5D).

### Ethmoid Nucleus (Eth)

The Eth stands out by its high neuronal density, the relatively large size of many of its neurons, and by its strong staining for PV and AChE (Figures 3F4, 6). The Eth is present in only a few coronal sections through the rostral third of APT and appears as an arch apposed to the ventrolateral surface of the PTL (Figures 1D, 6). It extends for 300–400 μm in the rostrocaudal dimension, and in some sections the distance between its dorsolateral and ventromedial ends reaches 700 μm. In most sections, the nucleus is 100–120 μm wide.

The neuronal density of the Eth is comparable to that of the APT or the SG (Figure 6A). The neurons of the nucleus are heterogeneous. Nevertheless, the most typical neurons tend to be multipolar or bipolar, with cell bodies 15–18 μm long and 9–10 μm wide that stain with cresyl violet darker than those of the medially located PTL and the laterally located Po. In some coronal sections, most neurons appear elongated, with their main axis parallel to the contour of the nucleus.

In sections processed for PV, the Eth exhibits strong immunostaining comparable to that of the APT (Figure 6B), in contrast to the paucity of staining in the PTL. Some Eth neurons are intensely immunoreactive and many dendrites are oriented parallel to the contour of the nucleus. The Eth is also highlighted by the dark staining of its neuropil with AChE (Figure 3F4). Accordingly, in PV and AChE material the ventral portion of the PTL appears sandwiched between APT and Eth. The Eth is immunonegative for CR (Figure 6C), and stains moderately for CB (not shown). No staining is seen in WFA material (Figure 3G4).

### Posterior Triangular Nucleus (PoT) and Posterior Nucleus (Po) of the Thalamus

The region of the posterior thalamus remaining among the nuclei described above consists of two continuous and ill-defined territories known as the PoT and the Po. The PoT, located more ventrocaudally, is surrounded by the MGBm laterally, the posterior intralaminar nucleus (PIN) ventrolaterally, and the PTL medially (Figures 1, 3). The more dorsal and rostral Po is limited laterally by the MGBd or the SG, depending on the level, medially by the PTL, and dorsally by LP (Figures 1, 3). The PoT and the Po contain a population of predominantly small and relatively scattered neurons and are not selectively highlighted in our material. For the purpose of our study, it is worth mentioning that wide regions of these two nuclei are immunoreactive for both CR and CB. More specifically, the narrow vertical stripe stained for CR and CB immediately lateral to the PTL may correspond to the medial border of the caudal PoT and Po (Figures 3D2,D3,E2,E3). Finally, these two nuclei exhibit moderate and diffuse labeling for PV and AChE (Figures 3B,F, 4A, 5A,C, 6B).

## Discussion

Our study provides useful morphological criteria to identify and delineate, with unprecedented precision, several nuclei of the posterior group of the thalamus of the rat. The application of a battery of complementary morphological techniques has been instrumental in pinpointing the features or combination of features that best particularize each nucleus (Watson et al., 2010). Major advances with respect to prior knowledge include: (a) the characterization of the PTL as a large and well-defined transitional nucleus with distinctive anatomical relationships; (b) the unequivocal location of the MGBm, whose proximity to the PTL must be taken into account to avoid spurious interpretations of scientific data; and (c) the demonstration that the SG extends for a considerable caudorostral distance and deviates progressively from the MGB. This information will be particularly useful in the case of the PTL, a nucleus for which very limited hodological and electrophysiological information exists. These and other aspects are discussed in the following sections.

### Pretectothalamic Lamina (PTL)

For a brain territory to be considered a separate nucleus or subdivision, it has to differ from its surrounding structures by at least three main criteria: basic structural organization (“architecture”), neural connections, and function (e.g., Kaas, 1982; Saldaña et al., 2007; Aparicio and Saldaña, 2014). The results of the present investigation strongly support the structural identity of the rat PTL. This conclusion is further strengthened by tract-tracing experiments from our laboratory, to be published separately, which ratify that the PTL is hodologically distinct (Márquez-Legorreta and Saldaña, unpublished observations).

The most conspicuous structural feature of the PTL is the abundance of myelinated fibers. Indeed the fibrillary border between the pretectum and the thalamus has been noticed in numerous studies carried out with a large variety of mammals. This fibrillary border has received varied names, including *tractus cortico-geniculatus to MGB* (Gurdjian, 1927 [rat]), *medullary lamina* (Burton and Jones, 1976 [rhesus monkey]; Hutchins and Weber, 1985 [squirrel monkey]), *internal medullary lamina* (Ingram et al., 1932 [cat]; Hirai and Jones, 1989 [human]; Lenz et al., 2010 [human]), *medial medullary lamina* (Jones, 1985 [primates, carnivores, tree shrews, lagomorphs, rodents and marsupials]; Jones and Hendry, 1989 [cynomolgus monkey and pigtailed monkey]), *external medullary lamina* (Oliver and Hall, 1978 [tree shrew], Jones, 2007 [primates, carnivores, tree shrews, lagomorphs, rodents and marsupials], Lenz et al., 2010 [monkey]), *lamina medullaris pretectothalamica* (Narkiewicz et al., 1985 [cat]; Słoniewski et al., 1985 [rat]; Słoniewski et al., 1987 [cat]; Moryś and Mamos, 1987 [rat]; Moryś et al., 1987 [cat]; Moryś et al., 1989 [primates, carnivores, insectivores, lagomorphs and rodents]), and *posterior limitans nucleus* (LeDoux et al., 1985, 1987 [rat]; Paxinos and Watson, 1986 [rat]). We suggest that this structure be known by the descriptive name *PTL*, a simplified English version of the Latin term *lamina medullaris pretectothalamica*, proposed by Moryś and his coworkers (Narkiewicz et al., 1985; Słoniewski et al., 1985, 1987; Moryś and Mamos, 1987; Moryś et al., 1987, 1989). Our proposed nomenclature eliminates the topographical confusion of previous names and clarifies the difference between the PTL and the classical nucleus limitans of the thalamus (see below). The adjective “medullaris”, used by the classical neuroanatomists to mean “fiber-rich”, is seldom utilized nowadays and may be a source of confusion due to its resemblance with the term “medulla”; we choose, therefore, not to incorporate this adjective in the name of the nucleus.

From the time the fibrillary border between the pretectum and the thalamus was noticed, several decades went by before it was recognized that the neurons embedded in this fiber bundle constitute a separate nucleus. These neurons have been described as predominantly small, with round, fusiform or triangular cell bodies (LeDoux et al., 1987; Moryś and Mamos, 1987; Gaillard et al., 2013) and having long, slender, sparsely branched dendrites oriented mostly parallel to the contour of the nucleus (Clerici et al., 1990; Winer et al., 2002, see their Figures 2D,E, 4G). Our data confirm these features in the rat and reveal that in the caudal portions of the nucleus the neurons tend to be multipolar and lack a conspicuous orientation.

A very effective tool to visualize the PTL of the rat is WFA staining, which highlights very strongly the caudal and ventral areas of the nucleus. This labeling consists of densely stained perineuronal nets, as well as diffuse labeling of the neuropil. The nature of PTL neurons surrounded by perineuronal nets remains unknown. WFA has been shown to bind preferentially to the perineuronal net of GABAergic neurons (reviewed by Sonntag et al., 2015). For example, most GABAergic neurons in the inferior colliculus of the guinea pig are surrounded by perineuronal nets (Foster et al., 2014; but see Fader et al., 2016, for discrepant data in the mouse). However, it seems very unlikely that the neurons of the PTL surrounded by perineuronal nets represent inhibitory neurons because GABAergic neurons are exceedingly rare in the auditory thalamus of rodents (Ottersen and Storm-Mathisen, 1984; Winer and Larue, 1996; Arcelli et al., 1997; Ito et al., 2011). Future studies using multiple fluorescent labeling may prove very useful to unravel the nature of PTL neurons highlighted by WFA staining. It will also be necessary to investigate the usefulness of WFA to identify the PTL of other species. For instance, while WFA does not stain the area of APT adjacent to the PTL of the rat, it stains rather homogeneously the APT of the monkey (Preuss et al., 1998; see their Figures 1F–H, 2G–I), thus hampering the identification of the PTL.

Recognition of the PTL is further facilitated by the use of other chemical markers. In our material processed for AChE, PV, CB or CR, weak or absent labeling in the PTL contrasts sharply with intensely labeled adjacent structures, thus confirming previous results in the rat (Paxinos et al., 1980, 1999a,b; Paxinos and Watson, 1982, 1986, 1998, 2007; Moryś and Mamos, 1987; Celio, 1990; Arai et al., 1991; Battaglia et al., 1992a; Winsky et al., 1992; Olucha-Bordonau et al., 2004). Similar observations have been made in other species (Graybiel and Berson, 1980 [cat]; Abramson and Chalupa, 1988 [cat]; Caballero-Bleda et al., 1991 [rabbit]; de Venecia et al., 1995 [rabbit]; Gutierrez et al., 2000 [*Macaca mulatta*]; Cruikshank et al., 2001 [mouse]; Kosmal et al., 2004 [dog]; de la Mothe et al., 2006 [marmoset]; Anderson et al., 2007 [guinea pig]; Jones, 2007 [ferret, his Figure 11.4B; cynomolgus monkey, his Figures 11.8]; Llano and Sherman, 2008 [mouse]; Olkowicz et al., 2008 [opossum]; Wong et al., 2008 [squirrel]; Lu et al., 2009 [mouse]; Hardman and Ashwell, 2012 [marmoset]; Lanciego and Vázquez, 2012 [macaque]; Giráldez-Pérez et al., 2013 [mouse]; Takemoto et al., 2014 [mouse]), suggesting that the low expression level of these markers in the PTL is phylogenetically conserved.

A survey of the literature points out other markers useful to locate the PTL. For example, *cytochrome oxidase* provides a staining pattern rather similar to that of PV (Manocha and Bourne, 1966 [squirrel monkey, their Figure 9]; Major et al., 2003 [squirrel, their Figure 9A]; de la Mothe et al., 2006 [marmoset], Anderson et al., 2007 [guinea pig]; Cant and Benson, 2007 [gerbil]; Jones, 2007 [cynomolgus monkey, his Figure 11.9]; Wong et al., 2008 [squirrel]; Anderson and Linden, 2011 [mouse]; Takemoto et al., 2014 [mouse]). In sections processed for *NADPH-diaphorase*, the PTL stands out by its mostly negative staining, although the contrast afforded by this technique is not as strong (Caballero-Bleda et al., 1991, 1992 [rabbit]; Bertini and Bentivoglio, 1997 [rat]; Paxinos et al., 1999a,b [rat]; Olucha-Bordonau et al., 2004 [rat]). On the other hand, the PTL stands out by the abundance of fibers and/or cell bodies immunoreactive for neuroactive substances such as *neuropeptide Y, enkephalin*, and *neurotensin*, which are absent in the neighboring nuclei (Moyse et al., 1987 [rat]; Morin and Blanchard, 1995, 1997, 2001 [hamster]; Borostyánkoi et al., 1999 [cat]; Borostyánkoi-Baldauf and Herczeg, 2002 [human]). Interestingly, these neuropeptide-containing fibers are found preferentially in the dorsal and rostral portions of the PTL, which are not stained by WFA.

The distribution of chemical markers, together with the limited information available about the neural connections of the PTL, suggests that the nucleus includes two different and complementary regions. The caudal and ventral portions of the PTL, which are revealed by WFA, may be involved in the processing of auditory information because they receive projections from the inferior colliculus (Kudo and Niimi, 1980; LeDoux et al., 1985, 1987), the nucleus of the bic (Kudo et al., 1984), and the cerebral auditory cortex (Hofstetter and Ehret, 1992; Budinger et al., 2000). On the other hand, the rostral and dorsal portions, which are rich in neuropeptide-immunoreactive fibers, are considered a component of the circadian visual system because they receive direct input from the retina and from the intergeniculate leaflet (reviewed by Morin and Studholme, 2014). These two regions of the PTL seem to correspond, respectively, to the “tail” and the “head” described in the PLi of hamsters and mice (Morin and Studholme, 2014).

It remains to be determined whether the PTL is a pretectal or a thalamic structure, i.e., whether it derives from prosomere 1 or from prosomere 2. Some authors have considered the PTL as a component of the pretectum (Morin and Blanchard, 1997 [hamster]; Gaillard et al., 2013 [Nile grass rat]). Morin and Blanchard (1997) argued that it is pretectal based on the fact that it does not project to the cerebral cortex. We note, however, that the PTL contains neurons that innervate the auditory cerebral cortex (Doron and Ledoux, 2000 [rat]; Budinger et al., 2006, 2008 [gerbil]; Budinger and Scheich, 2009 [gerbil]; Rubio-Garrido et al., 2009 [rat]; Ji et al., 2016 [mouse]), or the claustrum (Słoniewski, 1983; Słoniewski et al., 1986; Moryś et al., 1987). Moreover, the projections from the inferior colliculus and the nucleus of the bic mentioned above target heavily also other thalamic nuclei, but not pretectal nuclei. These facts suggest that the PTL is a thalamic nucleus. Future studies with molecular developmental markers may be useful to resolve this question definitively.

### Medial Division of the Medial Geniculate Body (MGBm)

Our results on the cytoarchitecture and chemoarchitecture of the MGBm of the rat confirm for the most part previous descriptions. The presence of large neurons that stain darkly with standard histological dyes makes this structure readily distinguishable (e.g., LeDoux et al., 1985, 1987; Clerici and Coleman, 1990; Winer et al., 1999; Jones, 2007). Moreover, the MGBm stains also for PV (Celio, 1990 [rat], Paxinos et al., 1999b [rat]; Budinger et al., 2000 [gerbil]; Cruikshank et al., 2001 [mouse]; Giráldez-Pérez et al., 2013 [mouse]).

An additional marker for the MGBm revealed in our experiments is WFA. The staining of the MGBm was rather unexpected because previous analyses of the distribution of perineuronal nets in the central auditory pathway stated that they were conspicuously absent in the MGB (Brückner et al., 1998 [opossum]; Friauf, 2000 [rat]; Fader et al., 2016 [mouse]). Although the reason for this discrepancy is unclear, it may reflect differences in the staining protocols, in the experimental species and/or in the criteria used to define the MGBm. A methodological explanation is consistent with unpublished results mentioned by Sonntag et al. (2015), which indicate that “the use of the antibody AB1031 which specifically detects aggrecan yields solid perineuronal net expression in medial parts of mouse MGB.” In any event, the unequivocal staining pattern observed in our experiments stresses the usefulness of the WFA to locate the rat MGBm. Moreover, the fact that WFA stains the MGBm less intensely than the PTL enhances the distinction between these two nuclei, particularly at caudal levels, where the distance between them is minimal.

The previously neglected proximity between the caudal region of the PTL and the MGBm deserves a separate comment. The medial region of the auditory thalamus has been the subject of numerous hodological, electrophysiological, pharmacological and behavioral studies. Because the PTL and the MGBm are so close, it becomes exceedingly difficult to ascertain the degree to which the results of past experimental manipulations aimed at the MGBm actually reflected the involvement of the MGBm, and not the spurious involvement of the PTL. This concern is aggravated by the fact that the function or functions of the PTL remain completely unknown and therefore the MGBm may have been attributed roles actually performed by the PTL. Extreme caution must be exercised in designing and interpreting future investigations centered in the caudal thalamus.

### Suprageniculate Nucleus (SG)

Confirming previous observations, our results indicate that the SG of the rat is readily recognizable due to its peculiar, well-known cytoarchitecture (LeDoux et al., 1985, 1987; Moryś and Mamos, 1987; Clerici and Coleman, 1990) and its conspicuous absence of staining for CR (Arai et al., 1991 [their Figure 2A]; Winsky et al., 1992 [their Figure 8]). The SG is a long and continuous nucleus that extends caudorostrally from the level of the caudal MGB to the level of the rostral pole of APT. The distance between SG and MGB increases progressively in the rostral direction; indeed, in sections of the rostral half of the nucleus, the SG is clearly separated from the MGB by the intervened Po. This observation is consistent with the location of the SG of the hooded rat (Takahashi, 1985 [his Figures 2A–C, 5]), and with the location of the “dorsal SG” described by LeDoux et al. (1987), and is further supported by the distribution of the projection from the deep layers of the superior colliculus (SC; Linke et al., 1999; Horie et al., 2013; our unpublished observations) and from the posterodorsal area of the auditory cerebral cortex (Kimura et al., 2004, [their Figure 6]). However, the location and extent of the SG revealed by our results strongly contradict the generally accepted scheme that places the SG closely apposed to MGBd at all rostrocaudal levels (e.g., Winer et al., 1999; Paxinos and Watson, 2007). Therefore, a judicious reappraisal of the properties and neural connections previously attributed to the SG seems warranted.

The specialized literature includes a scientific term closely related to SG: the “suprageniculate-limitans complex”. This concept, favored and discussed in detail by Jones (1985, 2007), emphasizes the anatomical vicinity and cytoarchitectural similarity between the SG and the classical limitans nucleus of the thalamus, and has been used mostly for the brain of carnivores and primates (Jones and Powell, 1971; Burton and Jones, 1976; Harting et al., 1980; Heckers et al., 1992). In rodents, however, the limitans nucleus has not been identified unequivocally (Jones, 1985, 2007; Moryś et al., 1989; Jones and Rubenstein, 2004; Watson et al., 2012), and it is not clear whether certain parts of the SG may be the homolog of the classical limitans. Several similarities in neural connections support the homology. Both the rodent SG and the limitans nucleus of other species receive projections from the deep layers of the SC (Niimi et al., 1970; Benevento and Fallon, 1975; Glendenning et al., 1975; Burton and Jones, 1976; Benevento et al., 1977; Partlow et al., 1977; Harting et al., 1980, 2001; Huerta and Harting, 1984; Hoshino et al., 2010; Baldwin et al., 2013). Moreover, both the SG (Heath, 1970; Guldin and Markowitsch, 1983; LeDoux et al., 1985; Linke and Schwegler, 2000) and the limitans (Mufson and Mesulam, 1984; Jones, 1985; Friedman and Murray, 1986; Clascá et al., 1997) are connected with the insular cortex. Finally, the basal ganglia are innervated by the rodent SG (Kubota et al., 1991; Yasui et al., 1991; Moriizumi and Hattori, 1992) and by the limitans (Heath and Jones, 1971; Jayaraman, 1985; Smith and Parent, 1986; Nakano et al., 1990; Steriade et al., 1997; Harting et al., 2001; Jones, 2007). However, the distribution of neurochemical markers in the SG differs considerably from that of the “suprageniculate-limitans complex”. While the SG is clearly immunonegative for CR, the complex exhibits a high to moderate density of CR-immunopositive neurons (Jones and Hendry, 1989; Fortin et al., 1996, 1998; Morel et al., 1997; Steriade et al., 1997; Cicchetti et al., 1998; Munkle et al., 1999, 2000; Gutierrez et al., 2000; Jones, 2007; Morel, 2007; Lenz et al., 2010; Maseko et al., 2013). Future studies are needed to clarify the homology between the SG and the classical limitans. It will be interesting to investigate whether the Lef1, Gbx2 and Cad-6 genes, which are expressed strongly in the “suprageniculate-limitans complex” of the monkey (Jones and Rubenstein, 2004), are also expressed in the thalamus of the rat.

### The Posterior Triangular Nucleus (PoT) and the Posterior Nucleus (Po)

Our experiments reveal one interesting piece of information about the PoT and the Po: in sections stained for CB and CR, there is a clear vertical band of labeling at the most medial and caudal part of these nuclei. Although this vertical band may be confused with the PTL (e.g., Arai et al., 1991), it is actually located immediately lateral to the PTL. This narrow territory seems to correspond to the area that receives projections from the spinal cord (LeDoux et al., 1987; Gauriau and Bernard, 2004). This hypothesis is enhanced by the specific expression of substance P in this area (Battaglia et al., 1992a) and by the fact that this neuroactive substance is depleted from the thalamus following a ventrolateral chordotomy of the spinal cord (Battaglia et al., 1992b). These data suggest that, unlike the PTL, the narrow vertical strip immunopositive for CB and CR receives spinal somatosensory information.

### Concluding Remarks

Our results confirm the structural complexity of the zone of transition between the pretectum and the thalamus. Each nucleus in this territory can be defined by a unique combination of morphological criteria, including cytoarchitecture and myeloarchitecture and the expression of molecular markers. Given that most of these nuclei are small and show unique anatomical relationships, the reliability of future studies will depend on the unequivocal identification of the components of this area. The information provided in this article should prove advantageous to those interpreting the results of experimental manipulations and can be used to improve the design of new investigations.

## Author Contributions

All authors had full access to all the data in the study and take responsibility for the integrity of the data and the accuracy of the data analysis. Study concept and design: ES. Acquisition of data: EM-L, JACH-J. Analysis and interpretation of data: EM-L, JACH-J, ASB, ES. Drafting of the manuscript: EM-L, ES. Critical revision of the manuscript for important intellectual content: EM-L, JACH-J, ASB, ES. Obtained funding: ES, ASB. Study supervision: ES, ASB.

## Funding

This work was supported by the Instituto de Salud Carlos III (grant PI10/01803), Ministerio de Economía y Competitividad (grant BFU2013-43608-P), Junta de Castilla y León (grants SAN126/SA28/09 and SA343U14) to ES, and by the NIH/National Institute on Deafness and Other Communication Disorders (grant RO1 DC-002266) to ASB. EM-L was the recipient of fellowship 217039 from the CONACyT of Mexico.

## Conflict of Interest Statement

The authors declare that the research was conducted in the absence of any commercial or financial relationships that could be construed as a potential conflict of interest.

## Abbreviations

AChE: acetylcholinesterase
APT: anterior pretectal nucleus
bic: brachium of the inferior colliculus
CB: calbindin
CR: calretinin
csc: commissure of the superior colliculus
DLGN: dorsolateral geniculate nucleus
Eth: ethmoid nucleus of the thalamus
fr: fasciculus retroflexus
Hb: habenula
LGN: lateral geniculate nucleus
LP: lateral posterior nucleus of the thalamus
MGB: medial geniculate body
MGB: dmedial geniculate body, dorsal division
MGBm: medial geniculate body, medial division
MGBv: medial geniculate body, ventral division
pc: posterior commissure
PIN: posterior intralaminar nucleus of the thalamus
PLi: posterior limitans nucleus of the thalamus
Po: posterior nucleus of the thalamus
PoT: posterior triangular nucleus of the thalamus
PTL: pretectothalamic lamina
PV: parvalbumin
SC: superior colliculus
SG: suprageniculate nucleus
VLGN: ventrolateral geniculate nucleus
VPM: ventral posteromedial thalamic nucleus
WFA: *Wisteria floribunda* agglutinin.
